# Effects of modelling studies on controlled drainage in agricultural land on reduction of outflow and nitrate losses–a meta-analysis

**DOI:** 10.1371/journal.pone.0267736

**Published:** 2022-04-28

**Authors:** Barbara Kęsicka, Rafał Stasik, Michał Kozłowski

**Affiliations:** 1 Department of Land Improvement, Environmental Development and Spatial Management, Faculty of Environmental and Mechanical Engineering, Poznań University of Life Sciences, Poznań, Poland; 2 Department of Soil Science and Land Reclamation, Faculty of Environmental and Mechanical Engineering, Poznań University of Life Sciences, Poznań, Poland; University of California Davis, UNITED STATES

## Abstract

A review with meta-analysis of outflow and nitrate loss reduction in controlled drainage (CD) vs conventional, free drainage (FD) was carried out in the study. Since the results of experimental field studies usually cover short periods of data collection, hence in this paper, meta-analyses were based on model studies that usually cover a longer time range. The databases Web of Science and Scopus were searched for eligible English articles, published until December 2020, that describe the quantity and quality of drainage water. The meta-analysis of outflow and nitrate loss reduction in CD vs FD using the mean difference (MD) with a confidence interval (CI) of 95%. The influence of each study was measured through heterogeneity, sensitivity analyses and publication bias using STATISTICA (version 13.3) for all analyses. Of the 107 works identified, 18 were finally included in the analysis based on established criteria required for an appropriate meta-analysis. In general the results indicate a reduction in average drainage outflow of 30.5% (MD = -71.26 mm; 95% CI, -103.49 –-39.04; p = 0.000) in arable land with CD in comparison to FD practice. In the case of nitrate load the reduction was 33.61% and in the drainage water there was lower content in CD practice by an average of 8.36 kg NO_3_ ha^-1^year^-1^ (95% CI, -9.93 –-6.79; p = 0.000). Subgroup analysis of two meta-analyses indicates that the results concerning these associations may vary with the calculated weight for each article, in which the number of years of study had the most significant impact.

## Introduction

Drainage systems, comprising ditches or subsurface drains, are important for agricultural production of the world’s cropland. Subsurface tile drainage, i.e. free drainage (FD), is widely practiced in productive agricultural land with poorly drained soils in different parts of the world [1–3]. Benefits of this management practice are increased crop productivity and improved economic returns for crop producers. The consequences of drainage water from drainage network systems have been identified as an important factor contributing to increasing nutrient loads, mainly nitrate-nitrogen (${NO}_{3}^{-}‐N$), ${NH}_{4}^{+}‐N$, total nitrogen (TN) and total phosphorus (TP) into surface receiving waters [4–6]. Globally, it is estimated that the losses of ${NO}_{3}^{-}$ from agricultural systems account for 19% of the total amount of N used worldwide [7].

Tile drainage plays a significant role in soil nitrogen losses from agricultural lands, thus affecting surface and groundwater and leading to deterioration of water quality, especially as pollutants originating from agricultural drainage outflow include sediment, nitrogen and also phosphorus, pesticides, pathogens, salts, trace elements, and dissolved organic carbon [8–13]. This threatens the hydrological environment due to the accumulation of nitrates in water sources and oxygen deficiency [14–16]. This is confirmed by long-term studies carried out in various catchment areas, which indicate that agricultural systems affect nitrate levels in river waters. The research concerns facilities located in other climatic zones, soil properties and different cultivation systems, which allows a greater understanding of nitrate losses to subsurface drainage [17, 18]. This is a particularly important issue from the point of view of the environmental risk related to nitrate losses from agricultural sources. Several management practices have been developed to reduce the nitrate loading from artificially drained agricultural areas, including controlled drainage (CD), controlled tile drainage (CDT), drainage water management (DWM) [19–22], denitrifying bioreactors (DBR) [23–25], free water surface flow constructed wetlands (FWS) [26–28], saturated buffer zones (SBZ) and integrated buffer zones (IBZ) [29–31], and drainage water recycling [32].

CD is a conservation practice, to artificially raise and adjust the level to which the water table in a tile-drained field is allowed to rise, using a water control structure near the outlet of a drain to adjust the effective outlet elevation. Also, it can reduce nutrient loss during wet periods by storing more water in the field. This drainage water management affects the hydrological changes in the field cycle, which, depending on the location and weather conditions, increases some or all of the following flow components: root zone water storage, seepage, surface drainage, plant uptake and evaporation [33, 34]. Another benefit is reducing losses of nitrogen (N) from agricultural subsurface drained fields to surface waters. The consequence is an increase in groundwater levels, and longer anaerobic conditions are created in the soil, ideal for denitrification [35–37]. This process involves microbial respiration under anaerobic conditions in which nitrates from the ecosystem return reactive nitrogen (Nr) to the atmosphere as N_2_ and N_2_O emissions [38]. This practice is one of the mitigation measures targeting nutrient losses from agricultural drainage systems’ water before it enters streams [39].

Most of the research related to CD practice has been tested and widely used in US and Canadian studies for more than two decades [35, 36]. DWM is gaining popularity in other countries of the world due to improved quality of water flowing from drained agricultural fields to surface water. The practice has been tested on research facilities in several countries including Lithuania [40], Sweden [41], Denmark [39], Italy [42], China [43, 44], Iran [45], India [46], Japan [47], and Egypt [48]. Unfortunately, the results of experimental field studies usually include a short measurement period of CD effectiveness. Therefore, on the basis of these measurements, model studies covering a longer time range are carried out.

Among the many studies on the influence of CD on the drainage water quality and quantity, there are disparities in the obtained results. Some of them show a positive effect while others show a negative or no effect of CD on the quantity and quality of drainage water. Hence, in order to comprehensively analyze the effect of CD on the quantity and quality of drainage water, which will be statistically confirmed at the same time, a meta-analysis can be used, especially since there are no such studies. Increasing the implementation of the practice provides more data measured from field studies, and modelled data assessing the impact of CD on the quantity and quality of water in the discharge of tiles from fields or small catchment areas. One of the soil-specific models used to support tile drainage research is DRAINMOD created by Skaggs et al. [34, 49, 50, 51]. DRAINMOD is one of the most widely used hydrologic models to simulate subsurface drainage systems. It simulates surface runoff, infiltration, evapotranspiration, subsurface drainage and seepage from the soil profile in response to given climatological conditions, crop rotation, soil type, and drainage system parameters and management [34]. The program is used to simulate the water table depth, drained outflow or nitrate-nitrogen in the drainage water of drained soils in different parts of the world, for present, future and past data. In addition, there are several models to assess the long-term impact of agricultural management and climate change on crop production and water quality [52]. In recent decades, the ability of water quality models in the root zone to study the impact of agricultural management practices on water quality and crop growth in places significantly different in terms of climatic and pedological conditions has been widely created and improved.

Due to the shortage of previous studies comprehensively analyzing model data on the effect of CD on the quantity and quality of drainage water, including statistical validation, we applied a meta-analysis for this purpose. In this study, we focused on comparing the results obtained for DRAINMOD model studies under CD vs FD conditions and its effect on reduction of outflow and nitrate losses of drained agricultural land. Therefore, we used meta-analyses to synthetically and also statistically indicate the effectiveness of CD use in quantitative and qualitative aspects of drainage outflow.

## Materials and methods

### Search strategy

This meta-analysis was conducted (18 January 2021) by an advanced search in the *Web of Science* (WoS) by the *ISI Web of Knowledge* published by Thomson Reuters and Scopus; the owner of the database is Elsevier. The search scope was developed using the symbol "*" and the advanced search was performed for words or instructions created by logical operators. Keywords searched included (“controlled drainage” AND “drainmod”) until December 31, 2020. The scientific documents are included in the database Web of Science Core Collection: *Science Citation Index Expanded* (*SCI-EXPANDED*). The meta-analysis was limited to articles published in English.

### Inclusion and exclusion criteria

For further analysis, several articles were rejected after an initial review of the summaries and titles. A model study was selected considering CD and FD and their impact on the amount of outflow and nitrogen loss from fields. Articles from field studies, as well as those which provided insufficient data to conduct reliable meta-analysis or were reproduced publications, were also excluded.

### Data extraction

A standard protocol for obtaining data and information on the basis of which eligible articles were established was prepared after consultation. The data collected included the name of the article, the name of the first author, the year of publication, the amount of outflow, the loss of N, the number of years of modelling, and additionally the texture of the soil, the crop, the amount of annual rainfall, the average temperature, and depth and spacing of the drainage. Disputes regarding the eligibility of the article for meta-analysis were resolved through a group discussion. The information and data were entered in the standard Microsoft Excel data extraction form (S1 Data).

### Weights for individual articles in meta-analysis

In comparison to field research modelling of CD and FD practices gives the possibility of analyzing many combinations of different tile drainage and soil parameters, weather conditions and future climate predictions as well as CD variants and so on. Hence the derived conclusions of model simulations after validations can result from greater amounts of data than those obtained from field research. Pooled mean difference weighting by a function of sample size was used in meta-analysis weights [53, 54]. The following calculation is the sample weight for an individual study:

$$W_{i}=\frac{n_{tx,i}∙n_{ctr,i}}{n_{tx,i}+n_{ctr,i}}$$ (1)

where *n*_*tx*,*i*_ = total number of modelled variants of CD, *n*_*ctr*,*i*_ = total number of modelled variants of FD. The number of variants *ntx*,*i* and *nctr*,*i*, represents the result of multiplying the number of: years, locations, experimental plots, drainage spacing, drainage depth, variants of CD application as well as soil types (S1 Data).

### Data synthesis and analysis

On the basis of the data from each article, the average value of outflows and nitrogen losses for CD and FD were calculated according to the number of variants. A mean difference (MD) with a confidence interval (CI) of 95% was used as a standard for measuring the relationship between CD and FD. Statistical heterogeneity was assessed in studies using Q and I^2^ and L’Abbé plots. Q is Cochran’s heterogeneity statistic usually computed by summing the squared deviations of each study’s estimate from the overall meta-analytic estimate, weighting each study’s contribution in the meta-analysis. I^2^ explains the percentage of total variation across studies that is due to heterogeneity rather than chance, and it is a helpful estimate to investigate the causes and type of heterogeneity. Based on the Q value, T^2^ is determined, defined as the estimator of the variance of the actual effects [55, 56]. A cumulative meta-analysis was performed taking into account the chronological effect of individual studies on the calculated overall effect and the error in its estimation varying over time after accounting for subsequent publications. A sensitivity analysis was performed for the change in cumulative effect due to the exclusion from the meta-analysis of individual studies as this will change the standard error. Wherever the results were heterogeneous, a random-effects model was used in the meta-analysis. In this selected statistical model, we assumed that the true effect may vary depending on the study. We chose this variant because the studies differed significantly in depth and drainage spacing parameters, duration simulation, modelling accuracy, soil and climate context, and cultivated plant species used. Subgroup analyses were carried out for possible causes of heterogeneity. Integrated estimates and associated CI of 95% were evaluated using forest plots as visualizations. Publication bias was evaluated qualitatively using funnel plots, Egger regressions, and the Begg–Mazumdar correlation test [57, 58].

Values of *p* < 0.05 were considered as valid for heterogeneity tests. For statistical analyses, STATISTICA software (version 13.3), the Set Plus module with meta-analysis and meta-regression, was used.

## Results

### Selected articles

**Fig 1:**
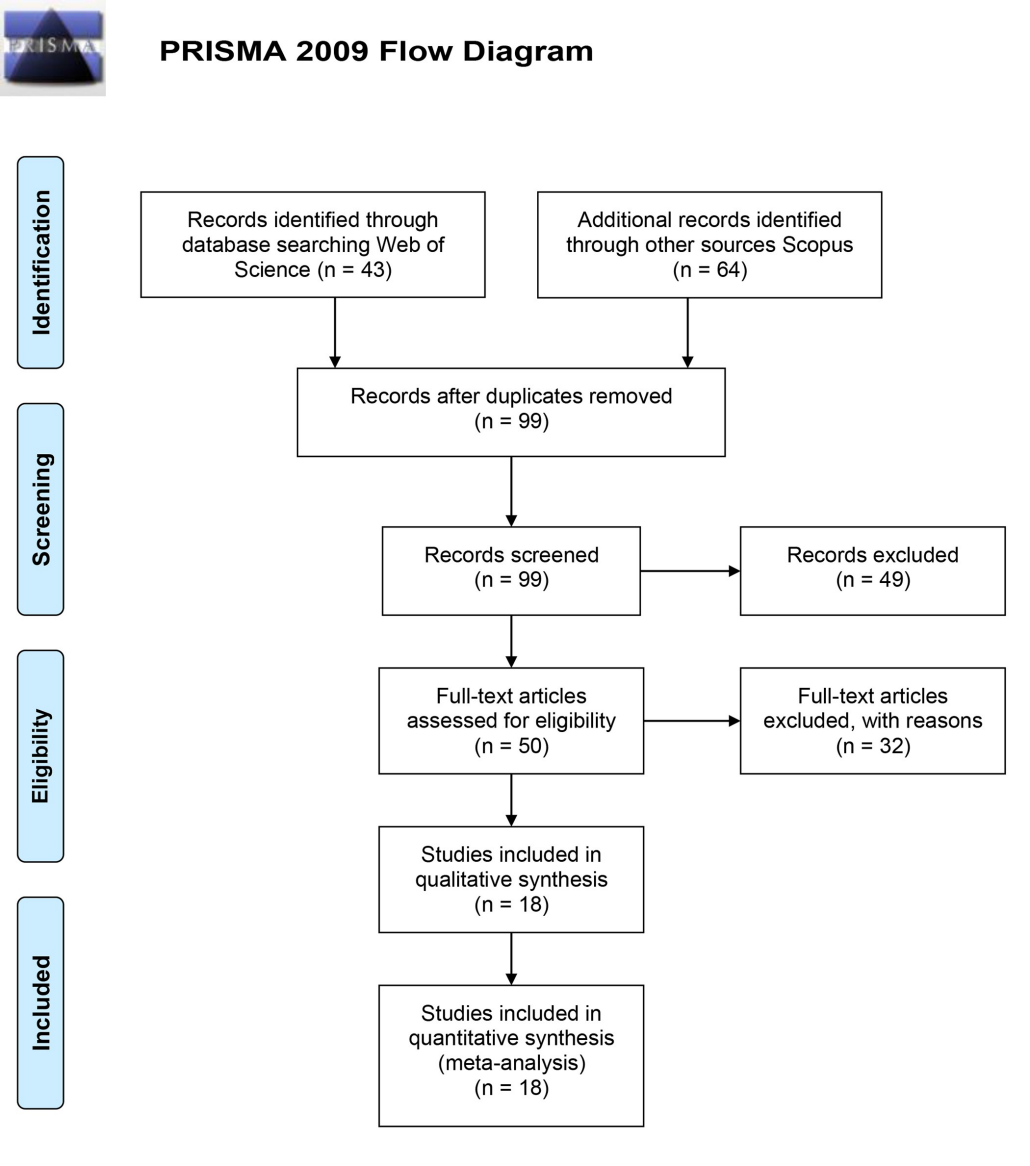
Flowchart of the literature search.

In an electronic search of the literature, 107 potential articles were identified; 43 were found in the WoS database and 64 articles in the Scopus database. Following the completion of the preliminary screening of abstracts and titles, 57 articles were excluded on the basis of the inclusion criteria (8 duplicate articles and 49 on the basis of unrelated titles and summaries) and 50 articles remained for a full review of the text. In the secondary study and after the full-text review, a further 32 articles were excluded; they were rejected due to missing information to create a meta-analysis data statement for outflows and nitrate losses. Ultimately, eighteen studies after these exclusions were selected for the final analysis (Fig 1).

### Description of selected articles

**Fig 2:**
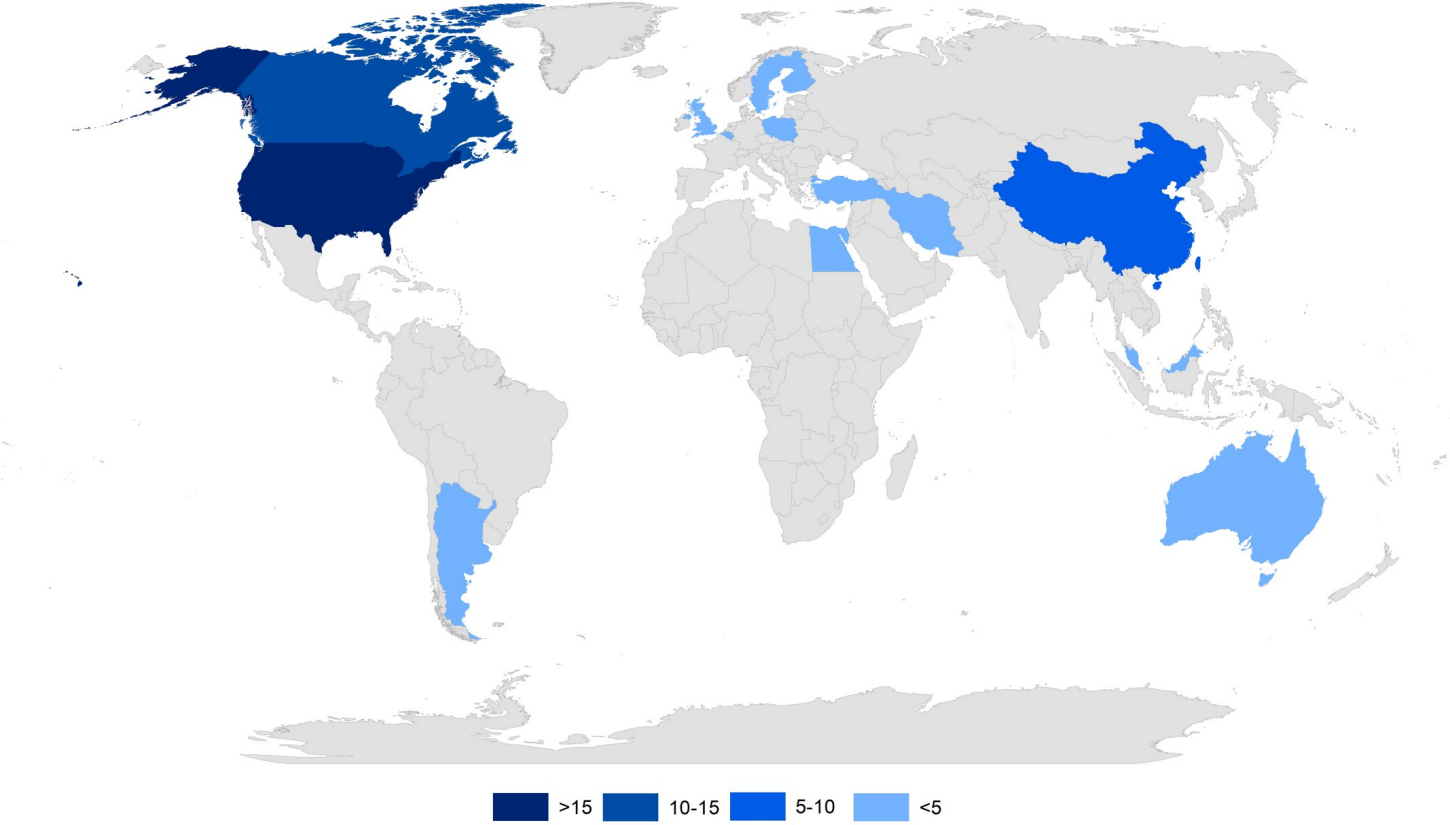
Countries to which the articles used in the record screening of articles relate.

The publications used in this meta-analysis were published between 1995 and 2020. Most of the articles were published in *Agricultural Water Management* (9 papers), *Transactions of the ASABE* (2 papers). One article each was published in 7 journals on the effects of CD on drainage water quantity and quality using DRAINMOD modeling results: *European Journal of Agronomy*, *Geoderma*, *Irrigation and Drainage*, *Journal of Environmental Quality*, *Journal of Soil and Water Conservation*, *Sustainability*, and *Transactions of the ASAE*. Of the selected articles, there are 10 (Skaggs et al. [59], Breve et al. [60], El-Sadek et al. [61], Ma et al. [52], Salazar et al. [62], Luo et al. [63], Skaggs et al. [64], Negm et al. [65], Negm et al. [66], Youssef et al. [67]) that are included in two meta-analyses of subsurface drainage outflow reduction and nitrate in drainage outflow loss. Subsequently, 4 publications (Skaggs et al. [68], Pease et al. [69], Sojka et al. [70], Singh et al. [71]) concerning outflows and 4 publications (Breve et al. [72], Singh et al. [73], Ale et al. [74], Ale et al. [75]) related to nitrogen losses were included in the relevant analyses. Most studies concern the modelling of outflows from research facilities located in the United States (83%), while the remaining 3 studies concern European countries: Belgium, Sweden and Poland (Fig 2). Table 1 presents the characteristics for selected articles regarding authors and year of publication, country, soil texture, years for which CD use was modelled, and depth and spacing of drains.

**Table 1:** Characteristics and information of studies evaluated in the meta-analysis.

Authors and date	Country	Soil texture	Years	Drain depth (m)	Drain spacing (m)
Skaggs et al. 1995 [59]	USA	sandy loam	13	1.00	10, 20, 30, 40, 50, 100
Breve et al. 1997 [60]	USA	sandy loam	2	1.25	23
Breve et al. 1998 [72]	USA	sandy loam	20	1.00	10, 15, 20, 25, 30, 40, 50, 100
El-Sadek et al. 2002 [61]	Belgium	sand	14	1.25	10, 25, 50, 100, 300
Ma et al. 2007 [52]	USA	loam	24	1.20	29
Singh et al. 2007 [71]	USA	silty clay loam	60	0.75, 1.20	10–50
Salazar et al. 2009 [62]	Sweden	loamy sand	3	0.99–0.83, 0.99–0.96	9
Luo et al. 2010 [63]	USA	silty clay	90	0.90, 1.20	9, 12, 15, 18, 24, 30, 36
Skaggs et al. 2010 [68]	USA	sandy loam	50	1.20	30
Ale et al. 2012 [74]	USA	clay loam	25	0.90, 1.20	12–25, 25–35, 35–45, 45–60, 60–80
Ale et al. 2012 [75]	USA	silty clay loam	10	0.90	10
Skaggs et al. 2012 [64]	USA	sandy loam, silty clay loam, clay loam	25	1.20, 1.25	30
Negm et al. 2016 [65]	USA	sandy loam	25	1.18	23
Negm et al. 2017 [66]	USA	silty clay loam, clay loam	1	1.30	36.5
Pease et al. 2017 [69]	USA	silty clay	30	1.14	10
Youssef et al. 2018 [67]	USA	loam	25	1.45	27.40
Sojka et al. 2019 [70]	Poland	sandy loam	3	0.90	7, 14
Singh et al. 2020 [73]	USA	silty clay loam	7	1.10	36.5

### Weights for individual articles

Due to differences in the basic characteristics of meteorological, soil and drainage networks introduced into models in different selected articles, a weight was established for each article. There is a significant difference in the years of modelling drainage in articles, from 1 to 90 years; it affected in some cases the final value of the weight. In addition, in some cases, the final assessment was also influenced by significant values in other parameters such as locations (Youssef et al. [67]), plots (Ma et al. [52]), spacing (Breve et al. [72]), and soils (Ale et al. [75]). Luo et al. [63] modelled for different spacing, others to determine outflows and others to determine nitrate loss. The range of weights for accepted articles is from 1 to 630. Three articles have the smallest weight values (Breve et al. [60], Salazar et al. [62], Negm et al. [66]) whose value reflects the number of years used for modelling. The largest weight values are published (Luo et al. [63], Youssef et al. [67]) with the largest number of modelling years and more variants of other parameters. The values of N_CD_ and N_FD_ for selected articles were used to determine the number of meta-analyses performed for the reduction of outflow drains and nitrate losses with outflow.

### Meta-analysis of drainage outflow and nitrate reduction

**Fig 3:**
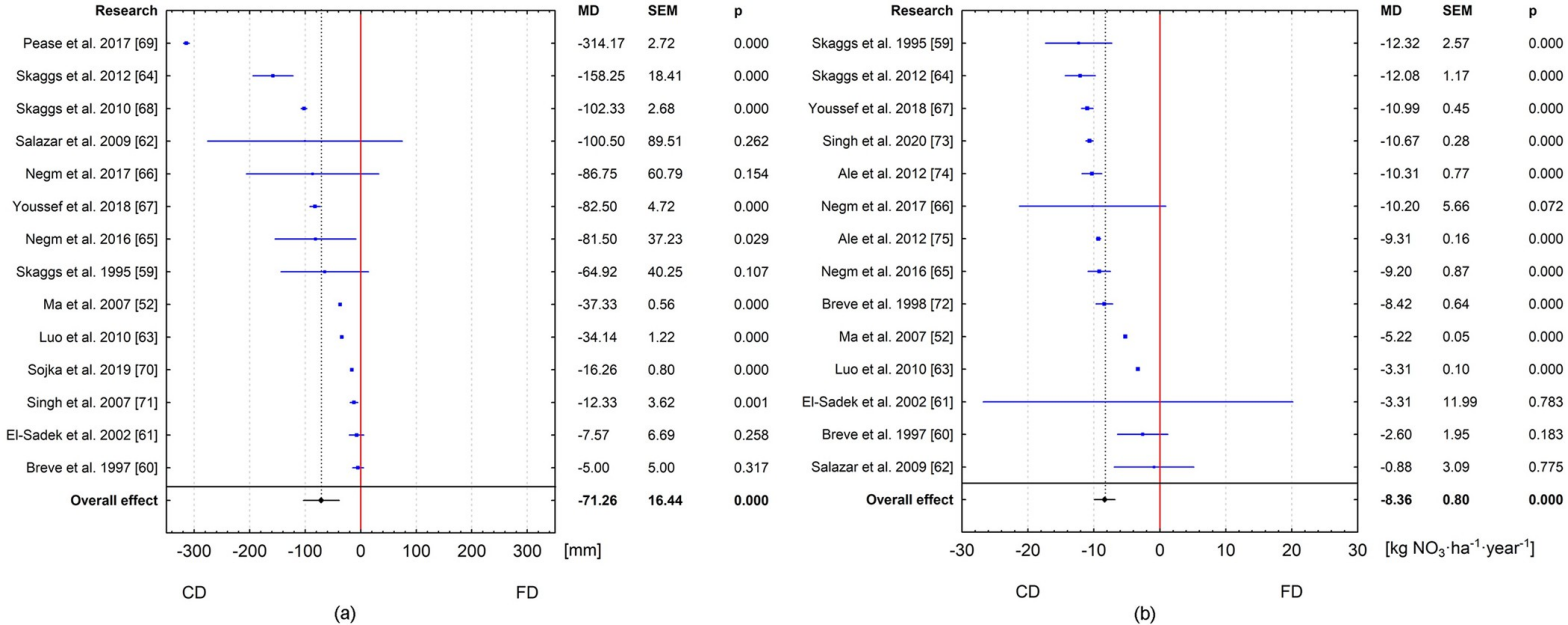
Meta-analysis for all articles: (a) subsurface drainage outflow reduction by CD versus FD; (b) reduction of nitrate load in drainage outflow by CD versus FD.

**Fig 4:**
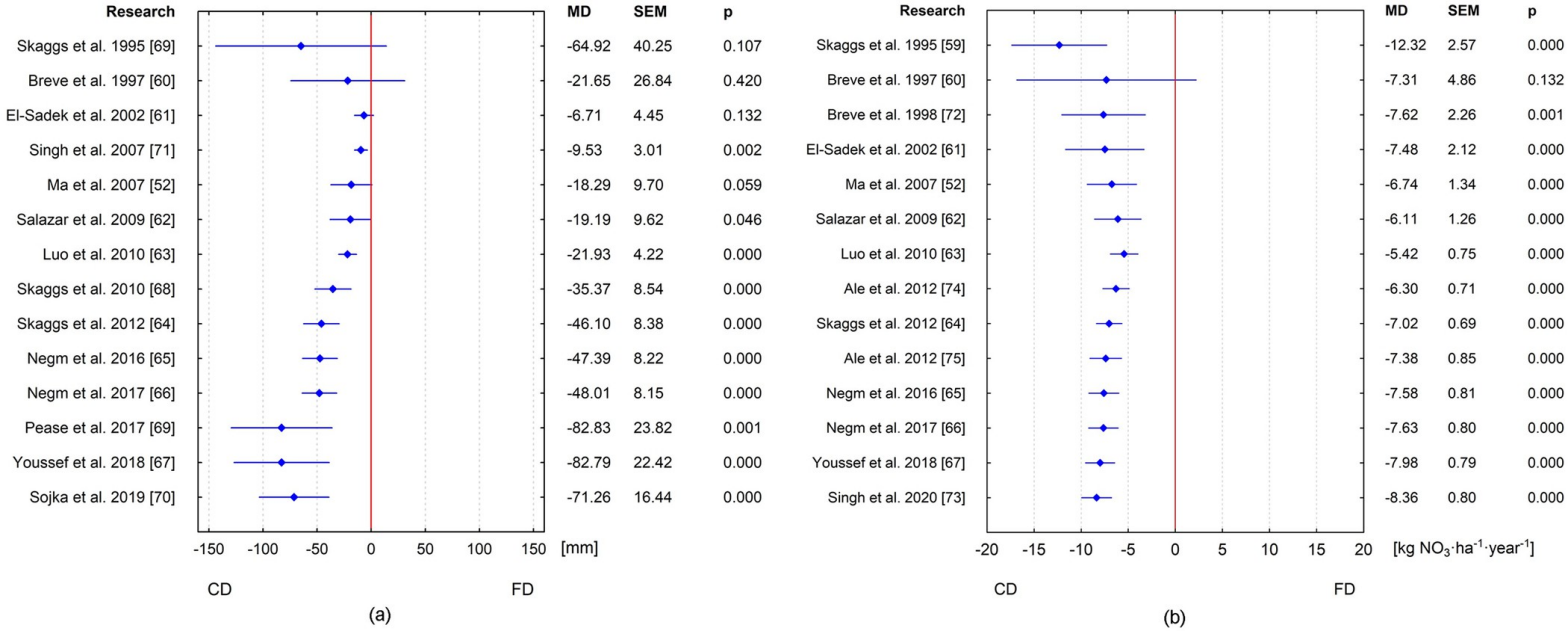
Cumulative meta-analysis: (a) subsurface drainage outflow reduction by CD versus FD; (b) reduction of nitrate load in drainage outflow by CD versus FD.

Two meta-analyses for subsurface drainage outflow reduction and nitrate in drainage water by CD versus FD were initially performed (Fig 3A and 3B). The first meta-analysis therefore used a random effect model to demonstrate how controlled drainage affects the reduction of drainage compared to conventional drainage. The overall effect from all articles analyzed indicates that the use of CD statistically significantly reduces drainage outflow by 71.26 mm per year. In absolute terms, this represents 30.5% of FD drainage outflow. All publications used in the meta-analysis indicated that the use of CD reduces outflow compared to FD. In this list, 5 publications were statistically insignificant (Fig 3A). Considering the year the study was published (Fig 4A), in the first period 1995–2007, when the first 5 publications were published, there was no statistically significant effect of CD on reducing drainage outflow. It is only with the inclusion of the 2009 study by Salazar et al. [62] that the effect was statistically significant and maintained after the inclusion of subsequent studies. Since this study, the 95% confidence intervals have narrowed significantly for the combined effect. The last 3 studies had high variability of results, which influenced the expansion of the 95% confidence interval for the combined effect.

The second meta-analysis also used a random-effects model to demonstrate how CD affects nitrate losses at low tide compared to FD. The findings of this analysis indicate a higher annual nitrate load in drainage waters in FD than in CD. The overall effect was statistically significant (Fig 3B) and showed an average reduction in nitrate losses of 8.36 kg NO_3_·ha^-1^year^-1^ (MD = -8.36; 95% CI: -9.93 –-6.79, p < 0.05) and this represents 33.61% of nitrate FD outflow. It is also apparent that in 4 out of 14 studies the results were statistically insignificant. The results of the cumulative meta-analysis indicate that from 1995 [59], each addition of subsequent studies (except study [72]) had a statistically significant impact on the summary effect for the cumulative analysis (Fig 4B). In general, each further addition of a literature item resulted in a significant narrowing of the 95% confidence interval for the summary effect.

**Fig 5:**
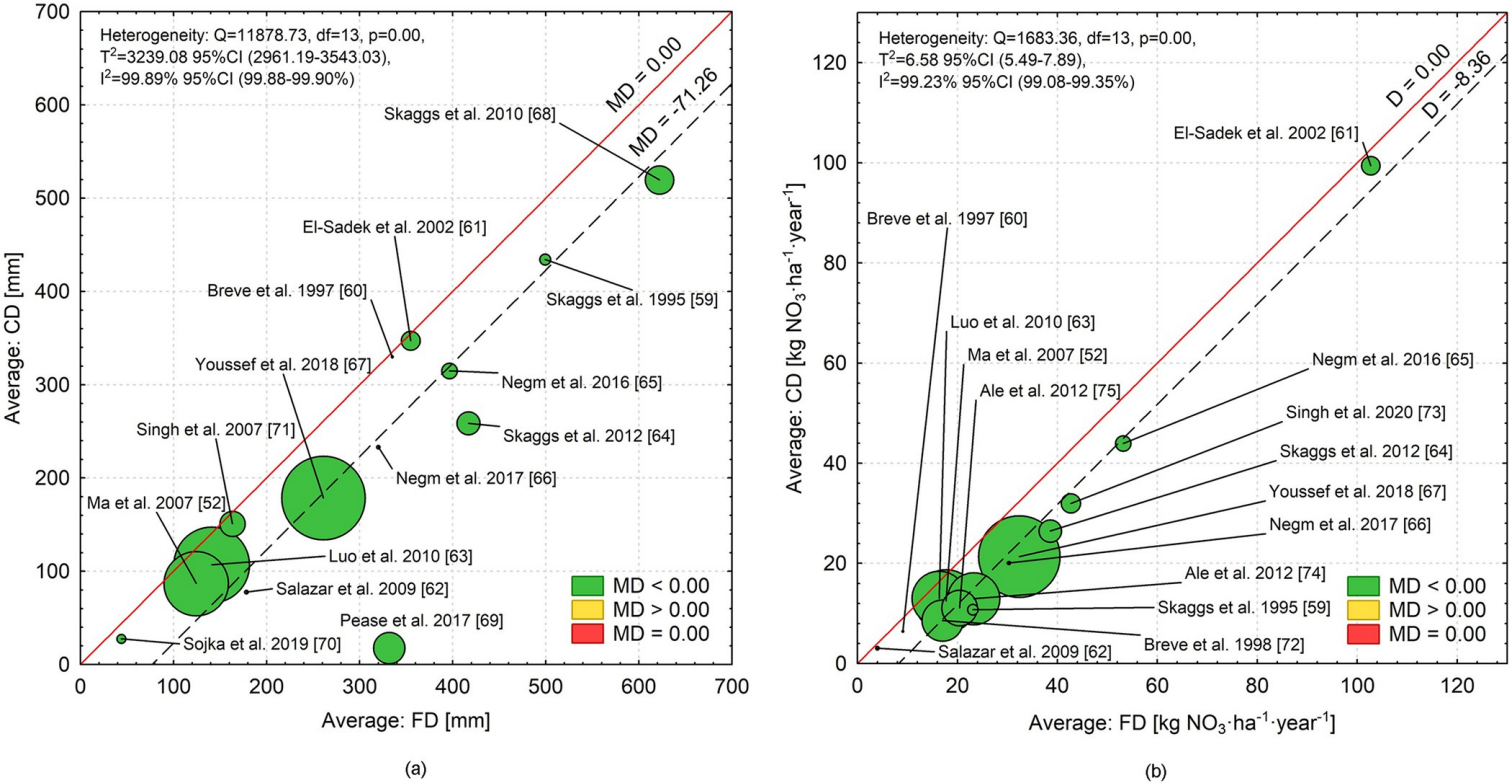
Heterogeneity analysis for all articles based on L’Abbé plot: (a) subsurface drainage outflow reduction by CD versus FD; (b) nitrate load in drainage outflow by CD versus FD.

Based on the heterogeneity analysis (Fig 5A), there was significant variation between studies analyzing the effect of CD on drainage outflow reduction (I^2^ = 99.89%; p = 0.000). The variance of T^2^ effects was 3239.08 and represented 99.89% of the observed variability. The relationship between the drainage outflow effect in CD and FD practice modelling was shown in the L’Abbé plot (Fig 5A). The solid line corresponds to the equilibrium level (MD = 0.00) and the dashed line to the overall effect determined in the meta-analysis (MD = -71.26 mm). The size of the markers is proportional to the contribution of a given study to the meta-analysis. A significant dispersion of points around the dotted line indicates high variability in the results of the research. The results of the meta-analysis indicate a positive correlation between drainage outflow in FD versus CD. The most far removed from the cumulative effect of the meta-analysis is the publication by Peace et al. [69], indicating a significantly higher average outflow value for FD and at the same time the greatest reduction in drainage outflow using CD. In three literature items [60, 61, 70, 71], the results indicated a small effect of CD on outflow reduction, of which two results of items [60 and 61] were not statistically significant. The outcome of study [68] is not much different from the combined effect of meta-analysis but has the largest drainage outflow values for both FD and CD. Analyzing the points (literature items except [69]) located within the solid and dashed lines, one can point out a certain regularity: the points are closer to the solid line the smaller the FD values are, while they move towards the dashed line as the FD increases.

Similarly, the results of the heterogeneity analysis were obtained for a meta-analysis of nitrogen losses, because Q is 1683.26 (df = 13) and the p value is less than 0.05. The variance of actual T^2^ effects is equal to 6.58 and represents 99.23% of the observed variability (I^2^ = 99.23%). In this case, the MD values of the individual tests are higher than the equilibrium level (MD = 0.00) (Fig 5B). In general, for nitrate in drainage outflow, the points (literature items) have less distribution around the dashed line than for water outflow. In addition, results from publications showing the lowest nitrate losses with both FD and CD were closer to the solid line (item [60, 62]), while those showing the highest nitrate losses with FD and CD (item [64, 65, 73]) were near the dashed line (except item [61]).

### Sensitivity and subgroup meta-analysis

A meta-analysis of subsurface drainage outflow reduction in current studies and sensitivity analysis were also conducted. After the sequential removal of each study from the analysis, the average overall effect changed. Thus, it was found that the most significant impact on the results was from the research article by Pease et al. [69]. After its exclusion, we would get a slightly larger average effect (MD = -47.48 mm), and the standard error for the overall effect would decrease by about 60%. The article had a strong impact on the results of the meta-analysis. This was the study [69] with the largest absolute reduction in drainage outflow among the articles analyzed (MD = -314.17 mm).

**Fig 6:**
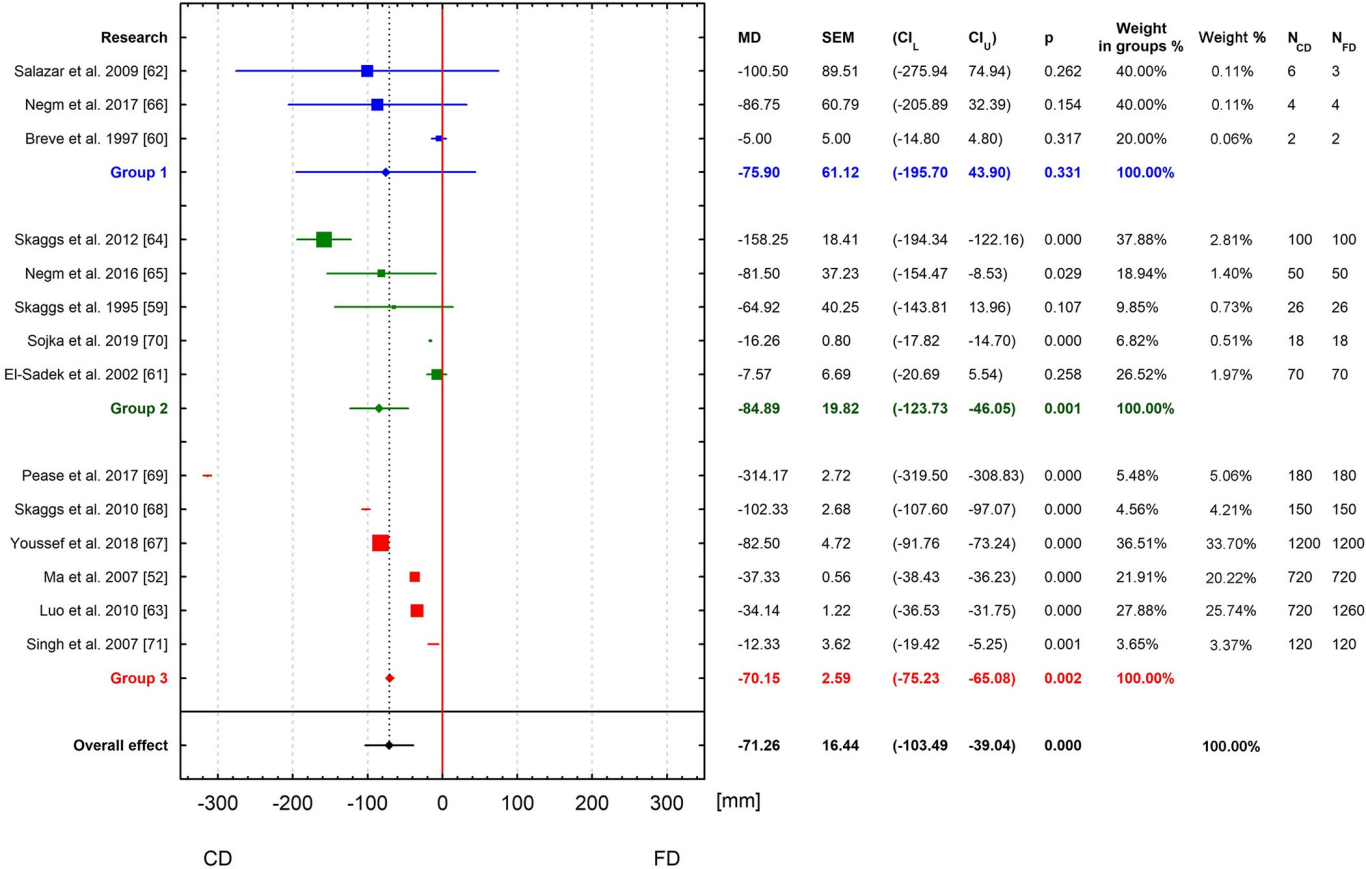
Meta-analysis in groups of subsurface drainage outflow reduction by CD versus FD.

Analyzed subgroups in the current meta-analysis take into account the number of years of model studies to minimize heterogeneity among different studies. Three groups are distinguished on the basis of the product of parameters used to calculate the weights of individual articles. The first includes studies up to 10 values of N_CD_ and N_FD_, the second from 10 to 100, and the third above 100. Fig 6 shows the results of the analysis in such defined groups. The first group’s overall effect score is statistically insignificant, MD = -75.90 mm (95% CI: -195.70–43.90; p = 0.331) and represents 33.73% of FD outflow. This group includes publications that analyzed the effect of CD on reducing drainage outflow for the fewest number of years. The final results of the overall effect for the second group, MD = -84.89 mm (95% CI: -123.73 –-46.05, p = 0.001) and the third, MD = -70.15 mm (95% CI: -75.23 –-65.08, p = 0.002) were statistically significant. These reductions represent 22.63% and 31.10% respectively. In addition, first and second groups contain publications (3 and 2, respectively) with an insignificant effect. When analyzing the forest plot for the distinguished groups the presented 95% confidence intervals for the group effect decrease from the first cluster to the third group. The closest to this effect is the D value of the third group, and from the selected articles the average difference between CD and FD for Youssef et al. [67] is 82.50 mm. The resulting total meta-analysis effect was most influenced by the three publications of Youssef et al. [67], Luo et al. [63] and Ma et al. [52], respectively 33.70%, 25.74% and 20.22%, in total explaining 79.66% of the results obtained. The smallest impact on the resulting total meta-analysis effect was found for the publications in the first group, two publications each of 0.11% and one of 0.06%. The most important in the first subgroup are two publications of 40% (Salazar et al. [62], Negm et al. [66]). The largest weights in the second subgroup are the two publications of Skaggs et al. [64] (37.88%) and El-Sadek et al. [61] (26.52%). In the third group, the three publications with the highest weights are those of Youssef et al. [67], Luo et al. [63] and Ma et al. [52], respectively 36.51%, 27.88% and 21.91%.

**Fig 7:**
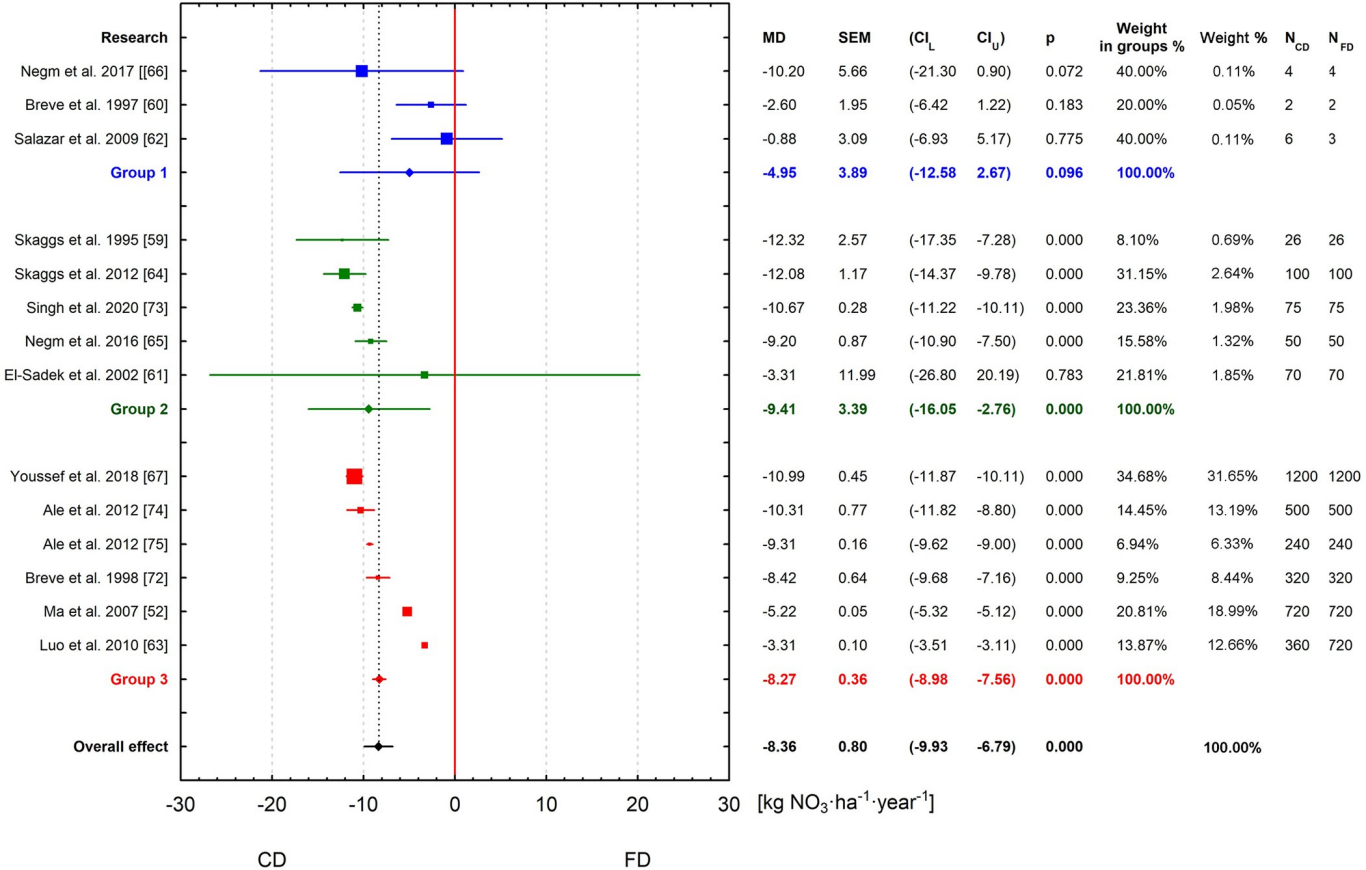
Meta-analysis in groups of nitrate load in drainage outflow by CD versus FD.

Likewise, three article groups were distinguished when analyzing the effect of CD on nitrate load reductions (Fig 7). For the first group, the mean effect score is statistically insignificant, MD = -4.95 kg NO_3_ ha^-1^year^-1^ (95% CI: -12.58–2.67; p = 0.096) representing a 28.18% reduction compared to FD. There was a statistically significant mean effect of CD practice on reduction of nitrate load for the second group, MD = -9.41 kg NO_3_ ha^-1^year^-1^ (95% CI: -16.05 –-2.76, p = 0.000), and the third, MD = -8.27 kg NO_3_ ha^-1^year^-1^ (95% CI: -8.98 –-7.56; p = 0.000), representing 23.19% and 34.59% respectively. This meta-analysis indicates that the overall effect of nitrate load in drainage is lower for CD by 8.36 kg NO_3_ ha^-1^year^-1^ (33.61%) compared to FD. In the second group is the article by Negm et al. [65], with a similar average value of 9.20 kg NO_3_ ha^-1^year^-1^. In the third group there is also a article by Breve et al. [72] with a mean nitrate load reduction effect (8.42 kg NO_3_ ha^-1^year^-1^) similar to that obtained in the meta-analysis. The total effects for the second and third groups are 1.05 kg NO_3_ ha^-1^year^-1^ higher and 0.09 kg NO_3_ ha^-1^year^-1^ lower, respectively, compared to the overall meta-analysis effect. In addition, a test based on Q statistics showed significant differences between the three test groups (p = 0.000). Also, in 4 (three of the first group and one of the second group) of the studies considered, the results were statistically insignificant for group effects. The resulting total meta-analysis effect was influenced the most by four publications: Youssef et al. [67], Ma et al. [52], Ale et al. [74] and Luo et al. [63], respectively 31.65%, 18.99%, 13.19% and 12.66%, together accounting for 76.49% of the results obtained. Here, the publications of the first group had the least impact on the total meta-analysis, two publications each of 0.11% and one of 0.05%, together affecting 0.27% of the final result. Two publications, by Salazar et al. [62] and Negm et al. [66], in the first subgroup are the most important, with 40%. In the second subgroup, the largest weight in the subgroup are three publications: Skaggs et al. [64] (31.15%), Singh et al. [71] (23.36%). In the third group, the two publications with the highest weights are those of Youssef et al. [67] and Ma et al. [52], respectively 34.68% and 20.81%.

### Publication bias

**Fig 8:**
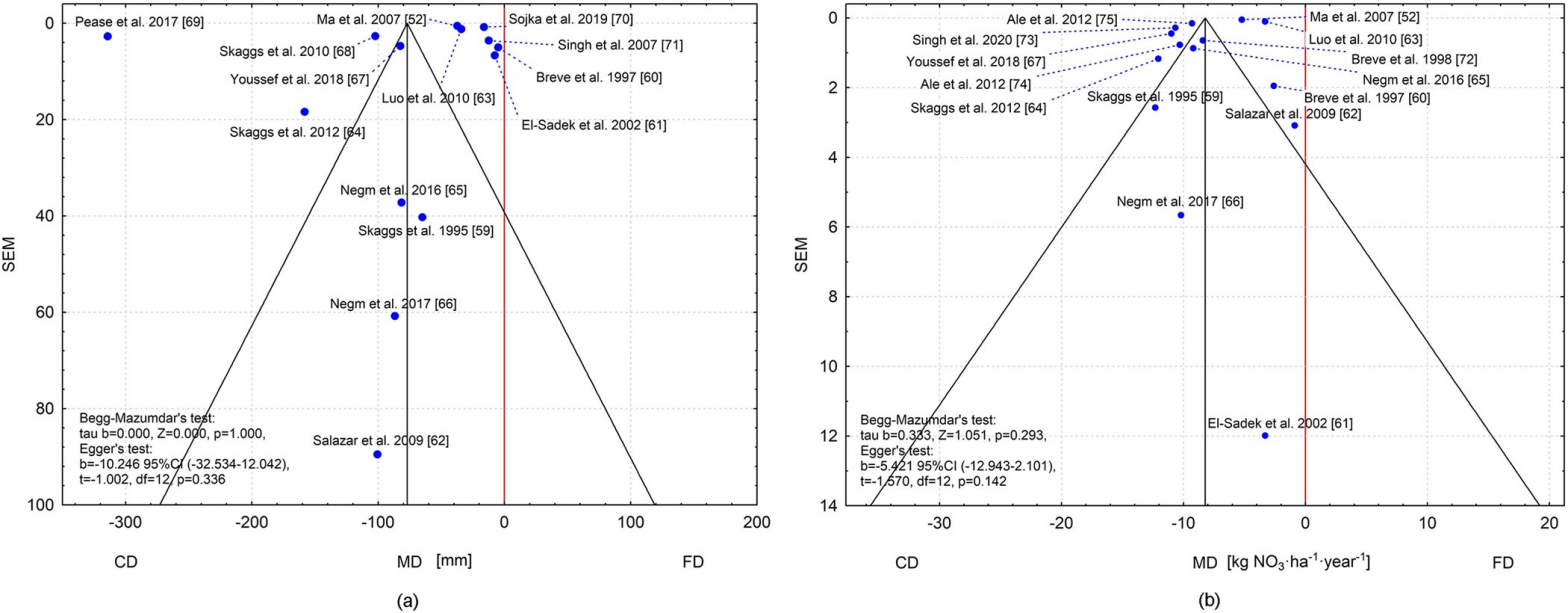
Funnel plot (from Begg-Mazumdar test) for publication bias: (a) subsurface drainage outflow reduction by CD versus FD; (b) nitrate load in drainage outflow by CD versus FD.

No signs of publication bias in the studies under consideration for two meta-analyses were shown by the funnel graphs presented in the Begg-Mazumdar test (Fig 8A and 8B). The Egger and Begg tests showed no evidence of publication bias for subsurface drainage outflow reduction and nitrate in drainage outflow by CD versus FD, respectively (p = 0.336 for Egger; p = 1.00 for Begg’s and p = 0.143 for Egger; p = 0.293 for Begg’s). In both graphs, a significant number of studies are at the top of the funnel outside the triangle, but they are close to the edge of the funnel. These are the studies with the smaller standard error and therefore have the strongest influence on the outcome of the analysis. In the funnel plot (Fig 8A), one study is outside the perimeter of the triangle (Pease et al. [69]); the only concern is the selection of this study.

### Limitations

The meta-analysis presented above has several limitations. Firstly, there were significant differences in the number of years of outflow and the nitrate reduction modeling for CD and FD between studies, which may affect average values. Hence, meta-analyses were conducted in groups. Secondly, due to insufficient data and statistical information in some studies, statistical values were calculated on the basis of the values given in the publications for each CD and FD variant. Publications were excluded if they did not contain data on the results of model studies. In the case of missing information, selected meta-analysis publications were not verified and consulted with their authors. Thirdly, this meta-analysis used mean outflow values and nitrate load in outflows in modelled studies. However, the input for each model differed among different studies. In addition, in meta-analysis, sample size and SD are particularly important in combining test results; this issue influenced our study. Furthermore, the effects of publication bias related to meta-analyses cannot be excluded.

## Discussion

In their project report from 2002 Abbott et al. [76] stated that CD has the potential to improve water use efficiency, maintain crop yields in periods of water stress, and ensure that land drainage systems work to the maximum benefit of farmers. Many articles supporting this thesis have appeared since this report was prepared.

**Fig 9:**
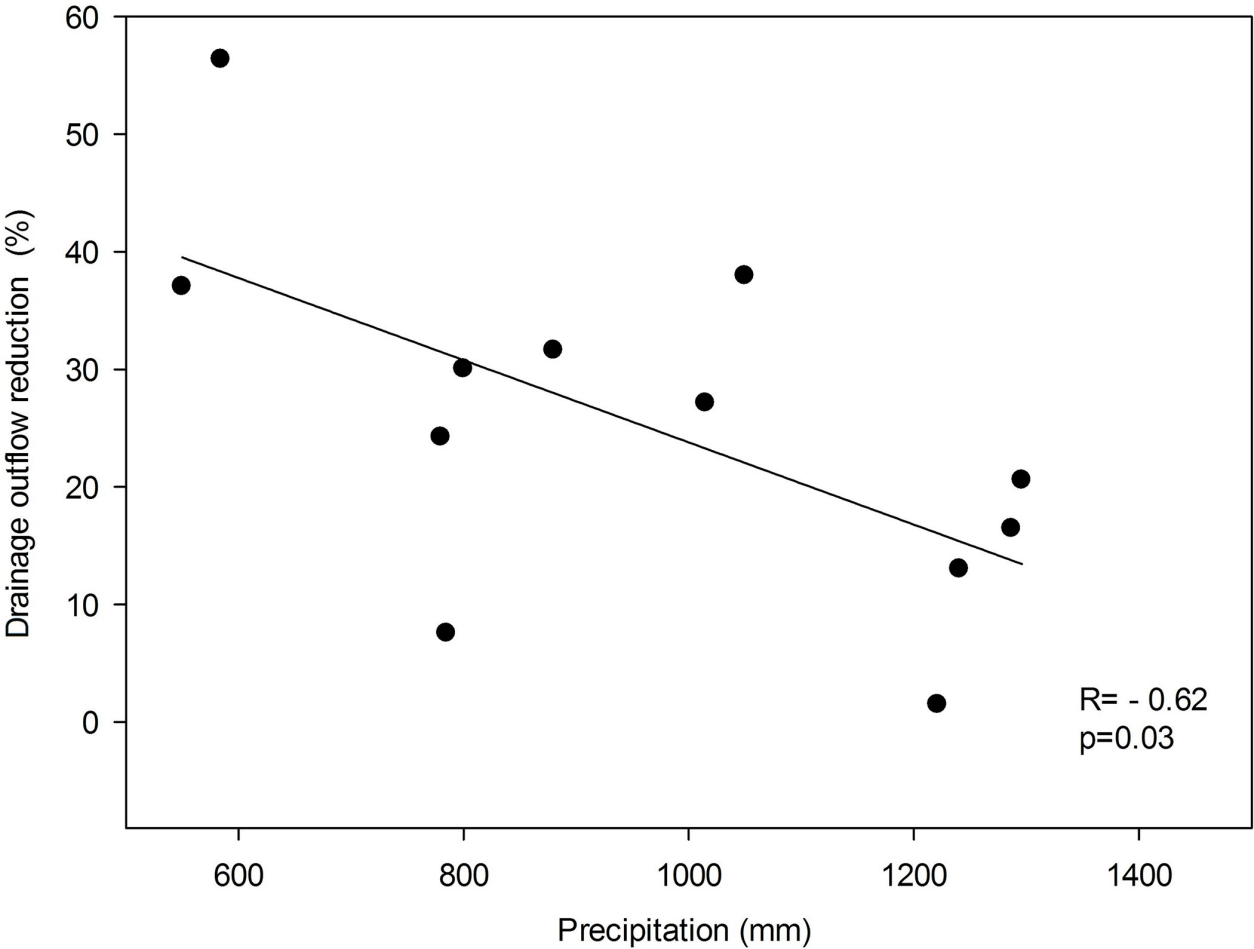
Reduction of drainage water with controlled drainage versus precipitation.

The current systematic study and quantitative meta-analyses found an average 30.5% reduction of drainage outflow in CD in comparison to FD practice, which averaged 71.26 mm in absolute values. Nevertheless, saving an average of about 71 mm of water by applying CD represents a significant amount of water considering global climate warming that can be used to mitigate adverse climate changes, including droughts. The present results are consistent with those of previous meta-analyses involving field and/or model studies. A meta-analysis presented by Wang et al. [77], including measured rather than simulated data, showed that the combined effect of CD on outflow volume reduction was 19.23%. However, it is worth noting that in this work [77] the combined result was based on both controlled surface drainage (ditches) and subsurface drainage (tile). Considering CD for subsurface drainage only, this effect was significantly higher at about 27.5%. Also according to the review conducted by Ross et al. [78] CD is an effective conservation practice for reducing drainage outflow. The results obtained [78] were significantly higher and CD reduced tile drainage volume by 46% on average. The authors also concluded that the greatest potential for CD to reduce discharge is during the non-growing season and confirmed that the efficiency of CD is especially influenced by drain spacing and management. Also Ale et al. [79] stated that there is reduction of drainage outflow using CD due to its principle of operation, but its effectiveness can vary significantly according to the different course of meteorological conditions, the strategy used, or the different parameters of the drainage network. Based on data from 1915–2006, Ale et al. [79] reported that CD has the potential to reduce annual drain outflow by 114 mm (52%), 94 mm (55%) and 75 mm 55%, respectively for 10 m, 20 m and 30 m drain spacings, relative to FD. Similar conclusions are found in some of the papers used in our meta-analysis [59]. For example, the highest outflow reduction using CD was found in the work of Pease et al. [69] and Salazar et al. [63], where outflow reductions of 95% and 61% were achieved at spacings of 10 and 9 m, respectively. On the other hand, the reductions reported by El-Sadek et al. [61] showed little effectiveness of CD in reducing outflow, averaging about 2.13%. In this case, it may have been due to the wide range of spacing adopted, from 10 to as much as 300 m (Table 1), and the fact that these simulations were carried out for soils with sand texture. The efficiency of drainage outflow reduction depends, among other factors, on climatic conditions, as pointed out by Wang et al. [77]. Based on the data and results in the articles selected for our meta-analysis, it can be concluded that there is a statistically significant negative relationship between total precipitation and reduction efficiency by CD expressed as a % (Fig 9). This suggests that the effectiveness of the CD system is higher for areas with lower precipitation. Such a relationship may be due to the fact that high precipitation amounts force the user to apply the FD practice more frequently to maintain optimal soil moisture.

In meta-analyses conducted in groups the results vary between 22.63% and 33.73% reduction of drainage outflow for CD practice compared to FD. In general, the meta-analysis of the subgroups indicates that as the weights became higher, the heterogeneity of drainage outflow reduction efficiency within the subgroup decreased and statistical significance increased. These weights were mainly determined by the number of study years.

**Fig 10:**
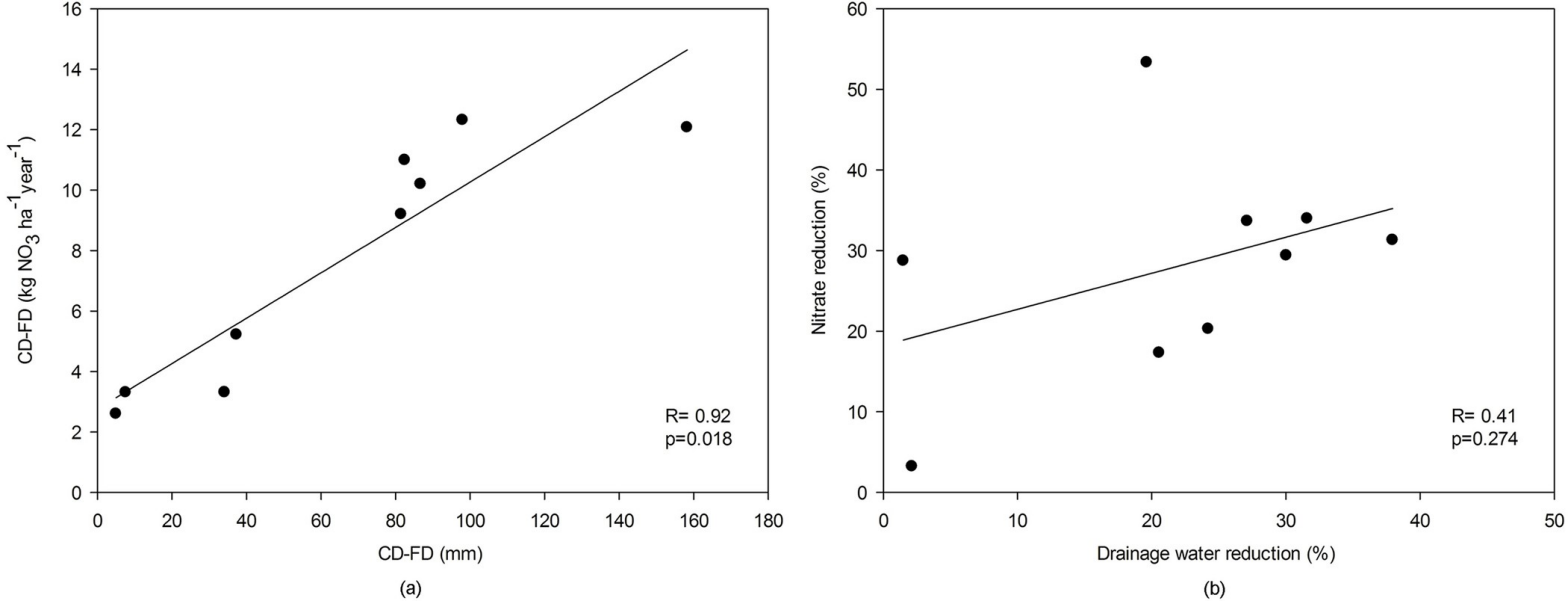
Absolute (a) and relative (b) reduction of nitrate versus absolute and relative reduction of drainage water with controlled drainage.

We also found using meta-analyses in the present paper that CD practice is an effective way of reducing nitrogen losses. According to our results, CD significantly reduced the nitrate loading of the drainage outflow. The average reduction in nitrate losses using CD was 8.36 kg ha^-1^year^-1^ compared to FD, and this was slightly lower than that obtained by Carstensen et al. [80] - 12.00 kg NO_3_ ha^-1^year^-1^ (Fig 3B). However, the heterogeneity of our meta-analysis results was relatively high, and the effectiveness showed a large dispersion around the mean (from to 0.88 kg NO_3_ ha^-1^year^-1^ to 12.32 kg NO_3_ ha^-1^year^-1^), which was not observed in previous studies [80]. This was expected, however, as the effect of CD on the efficiency of nitrate loss reduction depends on many factors, such as cropping system, drainage methods and control drainage management method, climatic and other conditions [43, 64, 77, 78], which varied among the literature items we analyzed. The absolute reduction of nitrate losses was mainly regulated by limiting the amount of water outflow into the drains, which was also emphasized in previous review studies [78]. This is confirmed by our results of the relationship between absolute reduction of drainage outflow and absolute reduction of nitrate expressed by the high value of the determination coefficient (R = 0.92 (Pearson), p = 0.018) (Fig 10A). The reduction in nitrate loading loss using CD solutions expressed in relative values (%) is on average 33.6%, with dispersion ranging from 3% to 53%. This average effect is comparable to that reported by Wang et al. [77] (36%) and lower than that reported by Carstensen et al. [80] (50%). In the case of the relationship between relative reduction of drainage outflow and relative reduction of nitrate losses, the correlation is not statistically significant (R = 0.41 (Pearson), p = 0.274) (Fig 10B), which does not confirm previous findings [80]. As with drainage outflows, in general, a negative correlation between increasing publication weights and heterogeneity within a subgroup and a positive correlation with statistical significance can be found for the meta-analysis of nitrate reduction efficacy subgroups. Since these weights were mainly determined by the number of study years (Table 1), it can be concluded that studies covering a longer period were more statistically significant than those conducted for shorter time intervals. Hence, the overall effect of CD on the reduction of nitrate loading loss in subgroup 1 was statistically insignificant. This first subgroup includes only 3 items of articles with a study period of only 2–3 years. In the other two separated subgroups, the articles were for a much larger number of years; therefore, in both subcluster 2 and subcluster 3, the subgroup effects of CD on reducing nitrate loading loss were statistically significant. For subgroup 2, one literature item [61] had statistically insignificant results for the subgroup effect, which was related to the high variation around the average. The high variable effect of CD on reducing nitrate loading losses in that article was related to drain spacing, which varied widely from 10 to 300 m. This large variation in drain spacing, according to previous findings [78], significantly affects the amount of nitrate leached with drainage water and thus the effectiveness of CD versus FD.

Although the meta-analysis results obtained indicate that CD statistically significantly reduces drainage outflow as well as nitrate loading in drainage water, there is high heterogeneity of study results within the articles used in our meta-analysis. This is due in part to the varying number of years covered by the studies, as well as factors highlighted in other papers [77, 78, 80]. Hence, we suggest that future studies evaluating the effectiveness of CD compared to FD should be based on measured or model data covering long time periods. While the latter are obtainable from calibrated and validated models, the former are expensive and time-consuming. In addition, modeling studies can be conducted for a wide range of factors affecting CD effectiveness, as well as at different scales [78].

## Conclusions

This review study, based on meta-analyses of the scientific literature available until December 2020, indicates that CD is an effective practice for reducing drainage outflows and nitrogen load losses on agricultural land. Overall, CD application reduced the amount of drainage outflow by 30.5%, averaging 71.26 mm in absolute terms. It should be noted, however, that not every application of CD will yield similar quantitative results of water saved. In general, studies covering a longer period of analysis have higher statistical significance for the meta-analysis result than studies conducted for shorter time intervals. The results of this study showed that in agricultural areas, the effect of CD on reducing drainage outflow in relation to FD is more effective where there is less precipitation. For nitrate, the application of CD solutions reduces nitrate load losses with drainage water by an average of 33.61%, which is 8.36 kg NO_3_ ha^-1^year^-1^ in absolute terms. However, the findings of the meta-analysis indicate that the heterogeneity of the results of the effect of CD on reducing nitrate losses with drainage water is very high (from to 0.88 kg NO_3_ ha^-1^year^-1^ to 12.32 kg NO_3_ ha^-1^year^-1^). It decreases with the increasing number of years included in the presented results in individual articles. The reduction of nitrate losses in absolute values was mainly regulated by the reduction of drainage outflows, while a weaker relationship is found between the relative reduction of nitrate losses and the relative reduction of drainage outflows.

## Supporting information

S1 Checklist (DOC)

S1 Data (XLSX)
